# Associations of Adenovirus Genotypes in Korean Acute Gastroenteritis Patients with Respiratory Symptoms and Intussusception

**DOI:** 10.1155/2017/1602054

**Published:** 2017-02-01

**Authors:** Jae-Seok Kim, Su Kyung Lee, Dae-Hyun Ko, Jungwon Hyun, Han-Sung Kim, Wonkeun Song, Hyun Soo Kim

**Affiliations:** Department of Laboratory Medicine, Hallym University College of Medicine, Hwaseong, Republic of Korea

## Abstract

Human adenoviruses (HAdVs) cause a wide range of diseases, including respiratory infections and gastroenteritis, and have more than 65 genotypes. To investigate the current genotypes of circulating HAdV strains, we performed molecular genotyping of HAdVs in the stool from patients with acute gastroenteritis and tried to determine their associations with clinical symptoms. From June 2014 to May 2016, 3,901 fecal samples were tested for an AdV antigen, and 254 samples (6.5%) yielded positive results. Genotyping using PCR and sequencing of the capsid hexon gene was performed for 236 AdV antigen-positive fecal specimens. HAdV-41, of species F, was the most prevalent genotype (60.6%), followed by HAdV-2 of species C (13.8%). Other genotypes, including HAdV-3, HAdV-1, HAdV-5, HAdV-6, HAdV-31, HAdV-40, HAdV-12, and HAdV-55, were also detected. Overall, 119 patients (50.4%) showed concomitant respiratory symptoms, and 32 patients (13.6%) were diagnosed with intussusception. HAdV-1 and HAdV-31 were significantly associated with intussusception (*P* < 0.05). Our results demonstrate the recent changes in trends of circulating AdV genotypes associated with gastroenteritis in Korea, which should be of value for improving the diagnosis and developing new detection, treatment, and prevention strategies for broad application in clinical laboratories.

## 1. Introduction

Human adenoviruses (HAdVs) have been associated with a wide range of clinical symptoms, including gastroenteritis, acute respiratory infections, conjunctivitis, hemorrhagic cystitis, and meningoencephalitis [1, 2]. AdVs are among the main pathogens detected in cases of acute viral infectious diarrhea, especially in children less than 5 years of age [3], and account for 1–31% of all cases of diarrhea in children [4].

AdVs, belonging to the family Adenoviridae and genus* Mastadenovirus*, are nonenveloped viruses that are 70–100 nm in diameter and have linear, double-stranded DNA enclosed by a protein shell (capsid). AdVs are grouped into 6 species (A–G) based on the antigenic variants of the capsid protein and can be further differentiated into 70 HAdV genotypes [5–7]. AdVs have 11 structural proteins, three of which are capsid proteins such as hexons, penton bases, and fibers. In addition, there are group- and type-specific epitopes on both hexons and fibers.

Among the various serotypes of AdVs, two serotypes from species F, AdV-40 and AdV-41, have been clearly associated with infantile diarrhea and are thus referred to as enteric AdVs [2]. Most studies on gastroenteritis caused by AdV have focused on AdV-40 and AdV-41, and most of the commercial AdV detection PCR kits can detect only these two serotypes. However, other types of AdV in stool have been reported in diarrhea patients [2], and recent studies on intussusception have revealed the clinical importance of other serotypes of AdVs [8, 9].

Therefore, we performed molecular genotyping of HAdV strains in stool specimens collected from patients with acute gastroenteritis in Korea from 2014 to 2016, with the aim of investigating the incidence of AdV gastroenteritis, the distribution of AdV genotypes, the types other than types 40 and 41 that might be associated with gastroenteritis, and the relationship between AdV genotypes and clinical symptoms.

## 2. Methods

### 2.1. Patient Samples and AdV Antigen Test

From June 2014 to May 2016, 3,901 fecal specimens were tested for the presence of AdV antigen using RIDASCREEN Adenovirus Antigen Test Kit (R-Biopharm, Darmstadt, Germany) in the laboratory of Hallym University Dongtan Sacred Heart Hospital, a 650-bed university hospital in Korea. This kit uses an enzyme-linked immunosorbent assay technique for detecting AdV in stool samples and the monoclonal antibodies reactive to the AdV-specific hexon protein and can detect most types of AdV [10]. A total of 254 specimens (6.5%) showed AdV antigen-positive results. Of these, 236 samples were subjected to AdV PCR and sequencing, and 18 samples could not be further analyzed due to an insufficient stool volume or missing samples. Stool samples were diluted to a 10% stool suspension in phosphate-buffered saline and stored at −70°C until used for AdV PCR and genotyping. The clinical data collected from the patients' medical records included their age, gender, concomitant respiratory symptoms (cough, sputum, rhinorrhea, pharyngeal injection, and paratonsillar hypertrophy), and intussusception. The age of patients with AdV antigen-positive results ranged from 0 days to 87 years; 220 samples (93.2%) were from patients less than 5 years old. This study was approved by the Institutional Review Board of Hallym University Dongtan Sacred Heart Hospital (IRB No. 2014-069).

### 2.2. AdV Genotyping and Sequencing

Viral DNA extraction from fecal suspensions for PCR and genotyping was performed using the QIAamp DNA mini Kit (Qiagen, Hilden, Germany) and the QIAcube platform (Qiagen). AdV hexon genotyping was performed by PCR and sequencing using a specified primer set (ADHEX1F/AD2) according to previous studies with few modifications [11, 12]. For DNA extracts that could not be amplified by this primer set, a different primer set (AD1/AD2) was used for PCR [12]. The PCR products were visualized by electrophoresis on an agarose gel and analyzed by DNA sequencing. The nucleotide sequences were analyzed using ABI Prism BigDye Terminator version 3.1 (Applied Biosystems, Foster City, CA, USA), and genotypes were confirmed using the NCBI BLAST server of the GenBank database.

### 2.3. Statistical Analysis

Positive rates of each genotype were compared to those of the total group or other groups using Fisher's exact test or chi-square test. Titers of AdV antigen (estimated from the optical density [OD]) of each genotype were compared to those of the total group using the Student *t*-test. The tests were considered statistically significant for *P* values < 0.05. MedCalc version 15 (MedCalc Software, Mariakerke, Belgium) was used for all statistical analyses.

## 3. Results

### 3.1. Monthly Distribution of AdV-Positive Cases

**Figure 1:**
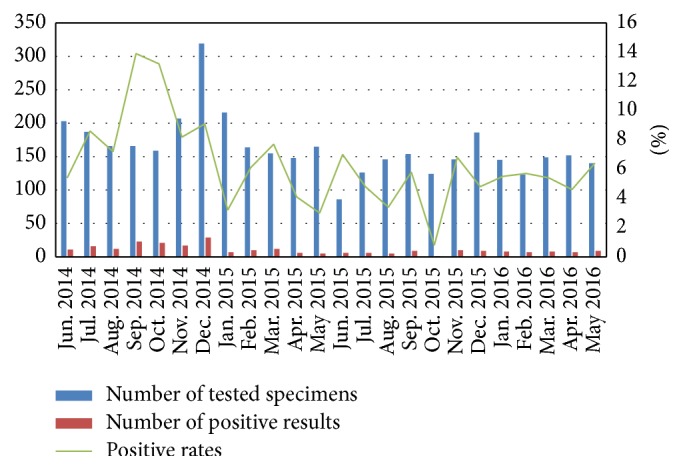
Monthly distribution of adenovirus infections in Korean patients with acute gastroenteritis detected by antigen enzyme-immunoassay test.

From June 2014 to May 2016, the highest positive rates among results over 10% from the AdV antigen test were observed in September and October of 2014 (13.9% and 13.2%), and the lowest positive rate was observed in October of 2015 (0.8%); the average positive rate was 6.5%. There was no seasonal peak detected, as positive AdV cases were observed throughout the study period (Figure 1).

### 3.2. AdV Genotype and Clinical Manifestations

The distribution of HAdV genotypes and their associations with clinical characteristics are summarized in Table 1. Of the 236 genotyped specimens, HAdV-41 was the most prevalent genotype, followed by HAdV-2. Other genotypes, including HAdV-3, HAdV-1, HAdV-5, HAdV-6, HAdV-31, AdV-40, AdV-12, and HAdV-55, were also detected. A total of 119 patients (50.4%) showed concomitant respiratory symptoms, and those infected with HAdV-2 (species C) showed significantly increased frequencies of respiratory symptoms (*P* < 0.01). Thirty-two patients (13.6%) were diagnosed with intussusception, including 9 of 14 (64.3%) patients infected with HAdV-1 (species C) but only 4 of 132 (3.0%) patients infected with HAdV-41 (species F). The rate of intussusception of other species C genotypes ranged from 26.7% to 50.0%, and the overall rate among patients infected with species C AdVs was 37.9%. In addition, 3 of 4 patients infected with HAdV-31 (species A) had intussusception.

### 3.3. AdV Viral Load according to Genotype

The average OD value of the enzyme-linked immunosorbent assay for detection of the AdV antigen (reflecting the viral load) was highest in the HAdV-41 and HAdV-40 groups, while the lowest viral loads were observed in the HAdV-3 and HAdV-55 groups (Table 1). The average OD value of AdV antigen was significantly increased in the HAdV-41 group and was significantly decreased in the HAdV-2 and HAdV-3 groups (*P* < 0.05) compared to the value for the total group.

### 3.4. Distribution of AdV Genotypes according to Age Group

Table 2 shows the age distribution of patients with genotyped AdVs. The frequencies of other types were not significantly different among different age groups.

## 4. Discussion

In this study, we genotyped AdVs identified in clinical stool samples from acute gastroenteritis patients in Korea. Few studies have examined AdV genotypes other than the most common types 40/41 in stool samples. In this study, HAdV-41 was the most prevalent genotype, which is an enteric AdV, although we also detected several other genotypes in our patients. These findings are similar to those previously reported [3, 13–17], with some interesting differences (Table 3). First, the C1 genotype was newly detected in Korean patients. Second, the prevalence of the C2 genotype was significantly increased compared to detection rates previously reported in a similar population during 2004–2006. Third, the B7 genotype was not detected in the present study. Finally, this is the first report of AdV B55 detection in stool samples, which is commonly known as a genotype associated with respiratory infection [18].

There are 70 AdV genotypes showing high genetic diversity, which has hindered the design of a single primer set for the detection of all genotypes. The two primers sets used in this study were proven to be suitable for detecting AdV-1, AdV-2, AdV-3, AdV-5, AdV-6, AdV-12, AdV-31, AdV-40, AdV-41, and AdV-55 in stool samples. We performed in silico analysis to confirm whether our primer sets could detect at least one of each HAdV type from HAdV-1 to HAdV-69, which showed less than four base-mismatches between our primer sequences and the 69 AdV types (data not shown). However, it was not clear whether this analysis predicted the successful amplification of the different strains, and the method used may fail at detecting variants with mismatches in primer-binding sites, despite the in silico primer set affinity results. Moreover, considering the high genetic diversity among AdVs, strains with single nucleotide variations at primer-annealing sites cannot be detected using these two primer sets. The majority of previous studies on the role of AdV in gastroenteritis have focused on the two main enteric genotypes (HAdV-40 and HAdV-41), and our study suggests that these detection methods are not suitable for identifying other genotypes. Although enteric AdVs are the most common cause of gastroenteritis, other genotypes can also be associated with enteric symptoms. Our results highlight the need to develop a more efficient set of primers for the detection of a wider range of genotypes, rather than restricting analyses to the most common types associated with certain symptoms.

In this study, the overall positive rate of the AdV antigen test in stool was 6.6%, and we did not observe a seasonal peak in the distribution of AdV gastrointestinal infections. Nevertheless, seasonal variation of gastroenteric illness linked to AdV infection remains a controversial topic, as peak incidences were reported in Korea (>10%) from August to September of 2004–2006 and in China in May and October of 2010 [13, 19]. However, no seasonal variation was detected in another study conducted in China during 2011-2012 [14].

To the best of our knowledge, no study has evaluated the association between the AdV antigen concentration in stool samples, the genotype detected, and concomitant respiratory symptoms. Patients infected with HAdV-40 and HAdV-41 had higher AdV antigen titers and a lower frequency of concomitant respiratory symptoms, which suggests that these types of AdV are primarily associated with gastroenteritis. HAdV-3 had a lower AdV titer and was significantly associated with respiratory symptoms, which suggests that AdV-3 is primarily associated with respiratory infections, and can be secondarily secreted into the stool. However, it is technically impossible to demonstrate a causative role for any AdV type other than those known to be enteric. It is possible that AdV types infecting the respiratory tract will be shed in the feces over prolonged periods of time [20, 21]. This applies to HAdV-C1, HAdV-B3, HAdV-C5, HAdV-C6, and HAdV-B55, especially if the patient has an active or has had a recent respiratory infection associated with one of these types. HAdV-A12 and HAdV-A31 have been reported to be associated with gastroenteritis, and therefore it is not surprising that their viral loads in stool samples were high (Table 2). Moreover, asymptomatic viral shedding in the stool from healthy children has been commonly reported [22, 23]. Therefore, caution should be taken when diagnosing adenoviral gastroenteritis based on the detection of AdV in stool.

In this study, 32 patients (13.6%) were diagnosed with intussusception, and the highest rate was observed in those infected with species C (37.9%). Other studies have described an association between AdV and intussusception [8, 9, 24]. In Australia during 2008–2011, HAdV species C was detected more frequently in cases than controls with 31/74 (41.9%) of cases testing positive compared to 39/289 (13.49%) controls [9]. In Thailand, all of the HAdVs detected (*n* = 12) in intussusception patients (*n* = 40) were reported to be of species C, and among the 44 intussusception patients identified in a study in Korea 22 (78.6%) had nonenteric AdVs, and AdV species C comprised the majority, with 20 cases (90.9%) [24].

## 5. Conclusions

In summary, HAdV-41 was the most frequent genotype isolated from patients with acute gastroenteritis in Korea in 2014–2016, and we found that other types of AdVs, which are known to be associated with respiratory infections, were detected in patients with acute gastroenteritis. We have expanded the list of AdV genotypes detected in stool samples and their association with respiratory symptoms and intussusception. Our results demonstrate the recent changes in trends of circulating AdV genotypes linked to acute gastroenteritis in Korea, which should be of value for improving the diagnosis and developing new detection, treatment, and prevention strategies for broad application in clinical laboratories.

## Figures and Tables

**Table 1:** Distribution of human adenovirus genotypes in stool specimens and their associations with respiratory symptoms and intussusception.

Adenovirus type	Number (%) of positive specimens	Titer (OD) of positive specimens (Mean ± SD)	Number (%) of patients with concomitant respiratory symptoms	Number (%) of patients showing intussusception
C1	14 (6.4%)	1.673 ± 1.284	8/14 (57.1%)	9/14 (64.3%)^a^
C2	30 (13.8%)	1.459 ± 1.172^a^	17/30 (56.7%)	8/30 (26.7%)
B3	21 (9.6%)	0.782 ± 0.794^a^	19/21 (90.4%)^a^	2/21 (9.5%)
C5	10 (4.6%)	1.563 ± 1.107	6/10 (60.0%)	3/10 (30.0%)
C6	4 (1.8%)	1.134 ± 1.370	3/4 (75.0%)	2/4 (50.0%)
A12	1 (0.5%)	3.500	1/1 (100%)	0/1 (0.0%)
A31	4 (1.8%)	1.836 ± 1.173	1/4 (25.0%)	3/4 (75.0%)^a^
F40	1 (0.5%)	2.744		
F41	132 (60.6%)	2.541 ± 0.933^a^	57/132 (43.2%)	4/132 (3.0%)^a^
B55	1 (0.5%)	0.360	1/1 (100%)	0/1 (0.0%)
Nontyped^b^	18 (7.6%)	1.169 ± 1.115^a^	6/18 (33.3%)	1/18 (5.6%)
Total	236 (100%)	2.009 ± 1.206	119/236 (50.4%)	32/236 (13.6%)

^a^Significant difference (*P* < 0.05) compared to the value for the total group.

^b^“Nontyped” indicates a failure in typing due to PCR or sequencing error (the chromatogram showed messy sequencing peaks or overlapping peaks) for genotyping.

**Table 2:** Distribution of human adenovirus genotypes according to patient age.

Genotype	0-1 yr	1-2 yr	2-3 yr	3-4 yr	4-5 yr	≥5 yr	Total	*P* value
C1	7	3	3	1			14	ns^a^
C2	14	10	2	3	1		30	ns
B3	4	5	3	3	3	3	21	ns
C5	7	3					10	ns
C6	3	1					4	ns
A12	1						1	ns
A31	3	1					4	ns
F40	1						1	ns
F41	43	46	21	11	7	4	132	ns
B55			1				1	ns
Nontyped	3	3	2		1	9	18	ns
Total	86	72	32	18	12	16	236	

^a^ns = not statistically significant.

**Table 3:** Comparison of results with previously reported adenovirus genotypes.

Country	Korea	Korea	China	China	Japan	Japan	Tanzania
Year of specimen collection	2014–2016	2004–2006	2011-2012	2010-2011	1995–2009	2009–2014	2010-2011
Number of typed specimens	236	113	219	31	Not presented	Not presented	37
AdV-C1	14 (6.4%)	0 (0.0%)^a^	Detected		Detected	Detected	3 (8.1%)
AdV-C2	30 (13.8%)	2 (1.7%)^a^	~13%	1 (3.2%)	Detected	Detected	3 (8.1%)
AdV-B3	21 (9.6%)	11 (9.7%)	Detected	7 (22.6%)	Detected	Detected	1 (2.7%)
AdV-E4		1 (0.8%)	Detected			Detected	
AdV-C5	10 (4.6%)			1 (3.2%)	Detected	Detected	
AdV-C6	4 (1.8%)		Detected				
AdV-B7	0 (0.0%)	5 (4.4%)^a^	12–15%				3 (8.1%)
AdV-B11			Detected				
AdV-A12	1 (0.5%)		Detected	2 (6.5%)			
AdV-B14			Detected				
AdV-A18							3 (8.1%)
AdV-D19							1 (2.7%)
AdV-A31	4 (1.8%)	2 (1.7%)	16–25%			Detected	5 (13.5%)
AdV-D37		1 (0.8%)					
AdV-F40	1 (0.5%)	3 (2.6%)	~11%	5 (16.1%)	Detected		10 (27.0%)
AdV-F41	132 (60.6%)	54 (47.8%)	38–46%	14 (45.2%)	Detected	71.3%	8 (21.6%)
AdV-B55	1 (0.5%)						
Nontyped	18 (7.6%)						
References	This study	[13]	[14]	[15]	[16]	[17]	[3]

^a^Significant difference (*P* < 0.05) compared to the frequency of the same adenovirus type observed in this study.
